# Association between lateral wall electrode array insertion parameters and audiological outcomes in bilateral cochlear implantation

**DOI:** 10.1007/s00405-022-07756-2

**Published:** 2022-11-27

**Authors:** Vivian Thimsen, Konstantinos Mantsopoulos, Tim Liebscher, Lava Taha, Felix Eisenhut, Heinrich Iro, Ulrich Hoppe, Joachim Hornung

**Affiliations:** 1 Department of Otorhinolaryngology, Head and Neck Surgery, Friedrich-Alexander-University Erlangen-Nürnberg (FAU), Waldstrasse 1, 91054 Erlangen, Germany; 2 Department of Neuroradiology, Friedrich-Alexander-University Erlangen-Nürnberg (FAU), Schwabachanlage 6, 91054 Erlangen, Germany

**Keywords:** Cochlear implant, Lateral wall electrode, Cochlear duct length, Frequency-to-place mismatch, Angular insertion depth, Speech recognition

## Abstract

**Purpose:**

The aims of this study were to compare speech recognition at different postoperative times for both ears in bilaterally implanted patients and to assess the influence of the time of deafness, frequency-to-place mismatch, angular insertion depth (AID) and angular separation between neighbouring electrode contacts on audiometric outcomes.

**Methods:**

This study was performed at an academic tertiary referral centre. A total of 19 adult patients (6 men, 13 women), who received sequential bilateral implantation with lateral wall electrode arrays, were analysed in retrospective. Statistical analysis was performed using two-sided *t* test, Wilcoxon test, median test, and Spearman’s correlation.

**Results:**

Postlingually deafened patients (deafness after the age of 10) had a significantly better speech perception (WRS65[CI]) than the perilingually deafened subjects (deafness at the age of 1–10 years) (*p* < 0.001). Comparison of cochlear duct length between peri- and postlingually deafened subjects showed a slightly significantly smaller cochleae in perilingual patients (*p* = 0.045). No association between frequency-to-place mismatch as well as angular separation and speech perception could be detected. There was even no significant difference between the both ears in the intraindividual comparison, even if insertion parameters differed.

**Conclusion:**

The exact electrode position seems to have less influence on the speech comprehension of CI patients than already established parameters as preoperative speech recognition or duration of deafness.

**Supplementary Information:**

The online version contains supplementary material available at 10.1007/s00405-022-07756-2.

## Introduction

Cochlear implants (CI) are high-tech tools that, through electrical stimulation of surviving hearing nerve fibres, replace the function of the inner hair cells in the cochlea and thus enable hearing in hearing impaired or even deaf patients. It is well-known, that inter-individual anatomical differences of the cochlea, such as length or the number of turns of the cochlear spiral, exist even among normally developed cochleae without any malformations [1–4]. Based on this fact and on the knowledge about the tonotopical properties of the cochlea, the assumption was made that both the position and insertion depth of the electrodes influence the audiometric results after cochlear implantation. The development of high-resolution imaging enabled exact examinations of the cochlea and more precise statements on tonotopical and anatomical conditions. In this context, Greenwood et al. [5] and Stakhovskaya et al. [6] provided the basis for further investigations concerning the CI electrode location with their studies of the frequency map of the cochlea. In the further course, various studies investigated the influence of possible location-related factors (e.g., angular insertion depth [AID], frequency-to-place mismatch [FPM] or angular separation) on the audiometric outcome in CI patients [7–14]. However, controversial opinions exist about the influence of the electrode insertion depth on speech recognition ranging from the significant benefit of deep insertion on speech perception [7, 8, 15, 16] to absence of any association or negative influence [9, 17–20].

As these previous studies show controversial results, the aim of our study was to assess the effect of these factors (FPM, AID, and angular separation between neighbouring electrodes) as well as of the time of deafness on audiometric outcomes and, in particular, to reconsider them intra-individually in a side-by-side comparison.

## Materials and methods

This study was performed at an academic tertiary referral center with specialization in cochlear implantation. Informed consent was obtained from each patient for diagnostic procedures, therapeutic measures and scientific data processing, approved by the University’s ethical review board and observing the university’s general contract conditions as well as the World Medical Association Declaration of Helsinki (as revised in 2013) [21].

A total of 19 adult patients (6 men, 13 women) underwent cochlea implantation on both sides. Therefore, in 8 patients the right ear and in 11 patients the left ear was implanted first. The median time interval between the two sides was 13 months (range 2–81 months). Pre- and postoperative audiometric measurements included pure-tone audiometry with evaluation of the four-frequency pure tone average at 0.5, 1.0, 2.0, and 4.0 kHz (4FPTA) for air conduction and speech recognition using the Freiburg monosyllable test, a phonemically balanced test consisting of 20 lists with 20 items each. In addition, the maximum Word Recognition Score (WRS_max_) with hearing aids was measured before surgery in free field in an anechoic booth. Each ear was tested separately in all measurements by masking the contralateral ear appropriately using headphones. The postoperative measurements with CI using the Freiburg monosyllabic words at 65 dB SPL (WRS_65_[CI]) were conducted at the first initial adjustment of the speech processor (4-week postop), 3 months, 6 months and 1 year after implantation. Furthermore, most of the patients had regular audiometric examinations with an average long-term follow-up period of 62.72 months (range 14–125 months). Three patients were excluded from the long-term follow-up analyses, as they had not received further audiometric measurements due to removal of the CI or severe dementia.

Every patient underwent a preoperative multislice computed tomography (MS-CT)—as well as postoperative panel computed tomography (FD-CT) of the temporal bone (Axiom Artis zeego, Siemens Healthineers, Erlangen, Germany). The images were further reviewed to determine the dimensions of the cochlea, cochlear duct length (CDL) and AID using OTOPLAN (CAScination, Bern, Switzerland and MED-EL, Innsbruck, Austria). As described previously AID was used to calculate the cochlear place frequency based on the spiral ganglion map (SG) [6].

FPM was determined by comparing the electrodes allocated frequencies (FAT-standard frequencies), which were adjusted 1 year after surgery at each individual electrode with the anatomical measured frequencies at the SG map for all 12 electrode contacts. Mismatch was then considered for the average frequency values of all electrodes and additionally for the average frequency of electrodes 5–8 (ca. 858–2274 Hz) corresponding to the most important frequency information for speech recognition [12]. Angular separation of neighbouring electrodes was calculated between the electrode contacts located in the 1–2 kHz region on the SG map (approximately 224°–333°) as previously described [11]. Audiometric values and imaging-associated values were compared for both ears intra-individually and inter-individually within the two groups of post- and perilingual hearing loss (deafness after the age of 10 or between the age of 1 and 10).

Statistical analysis was performed using two-sided *t* test, Wilcoxon test, median test and Spearman’s correlation depending on the analytical question. Scatter plots were created from the data. The software IBM SPSS statistics version 28 for Windows was used for the analysis. A *p* value of < 0.05 was considered statistically significant.

## Results

A total of 19 adult patients (6 men, 13 women, male to female ratio 0.46:1, mean age 54 years [range 18–75 years]), who received sequential bilateral implantation with lateral wall electrode arrays by the same surgeon, were analysed retrospectively. 9 electrode arrays “Standard” with a stimulation area of 26.4 mm and 29 “flex28” (76.3%) with a stimulation area of 23.1 mm (both MED-EL GmbH, Innsbruck, Austria) were implanted. The electrode array was inserted either via round window (RW; *n* = 35; 92.1%) or—if anatomically not possible—by basal cochleostomy (BC; *n* = 3; 7.9%). To prove the device function, intraoperative impedances and electrically evoked compound action potentials (ECAPs) were conducted. 11 patients suffered from postlingual (57.9%) and 8 from perilingual hearing loss (42.1%). The average time between the first and second implantation was 22 months (range 2–81 months).

The preoperative MS-CTs showed properly developed cochleae without malformations in all study patients. All twelve electrode contacts were anatomically inserted in all cases. Two cases of electrode kinking were detected in our study cohort (5.3%, both with “Standard” electrode arrays). All the other electrode arrays were properly located due to imaging results. In 33 ears all 12 electrodes were activated for auditory stimulation after 1 year, in four cases one electrode was inactivated [electrode 12 (*n* = 3), electrode 1 (*n* = 1)] and in one case two electrodes were inactivated [electrodes 11 and 12] due to discomfort or non-auditory sensations. Electrode deactivation had no significant influence on long-term speech perception (overall group: *p* = 0.377, *t* test two-sided; perilingual: *p* = 0.583, *t* test two-sided; postlingual: *p* = 0.347, *t*-test two-sided). No electrode change/reimplantation was necessary in the postoperative course.

### Postlingual versus perilingual deafness and speech recognition

**Fig. 1:**
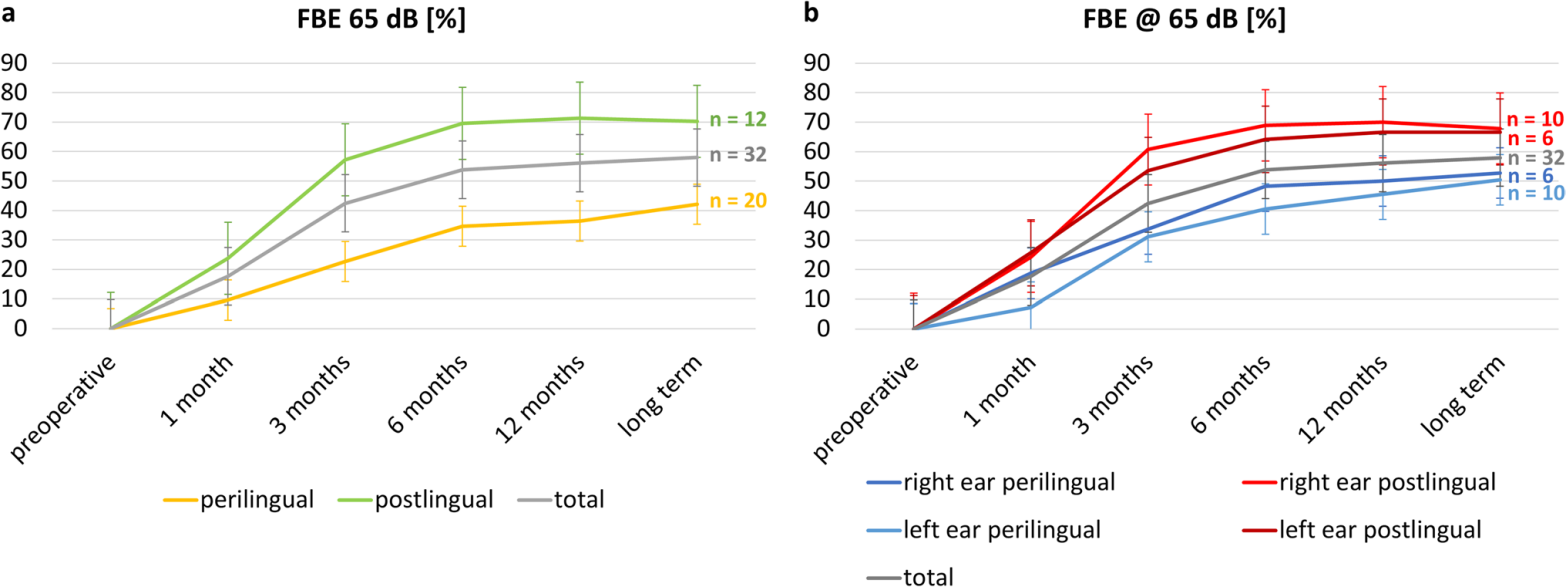
Development of speech perception after CI surgery. Comparison of WRS65 (word recognition score at 65 dB) scores between perilingual (yellow) and postlingual (green) CI recipients (**a**) and additionally between the right (red) and left (blue) ear (**b**) among the entire study cohort as a function of time. Mean values are indicated with the grey line. Three patients were excluded from analysis as we had no audiometric long-term values

The speech recognition results are plotted in Fig. 1 for WRS_65_ (in percent correct) as a function of time with an individual representation of peri- and postlingual patients (Fig. 1a) as well as the right and left ear (Fig. 1b). Concerning speech perception, postlingually deafened patients had a significantly better WRS_65_[CI] with 70.3% (> 1-year follow-up) than the perilingually deafened subjects (WRS_65_[CI] = 42.1% after > 1-year follow-up, *p* < 0.001 (*t* test for independent samples, two-sided). The study, furthermore, revealed a difference in the audiometric outcome of both ears in favour of the right side, but without achieving statistical significance (WRS_65_[CI-right, > 1 year] = 59.4%; WRS_65_[CI-left, > 1 year] = 56.6%; *p* = 0.726). This could be explained using the “ear effect” as described previously [22, 23].

### Association of several factors with speech perception

To prove anatomical differences in the subjects and sides, CDL was measured separately for both sides as described earlier [24, 25]. Average values for right and left CDL showed no significant differences compared between individuals (right: 36.53 mm; left: 37.92 mm; *p* = 0.132; *t* test for independent samples, two-sided). Comparison of CDL between peri- and postlingually deafened subjects showed a slightly significantly smaller cochlea in perilingual patients (mean_peri_ = 36.23 mm; mean_post_ = 37.95 mm; *p* = 0.045). In addition, matching earlier reports [26] the CDL was significantly larger in male (mean_male_ = 39.39 mm [Range 35.67–44.51]) than in female patients (mean_female_ = 36.23 mm [Range 32.79–42.91 mm]) (*p* = 0.001, Mann–Whitney *U* test).

**Fig. 2:**
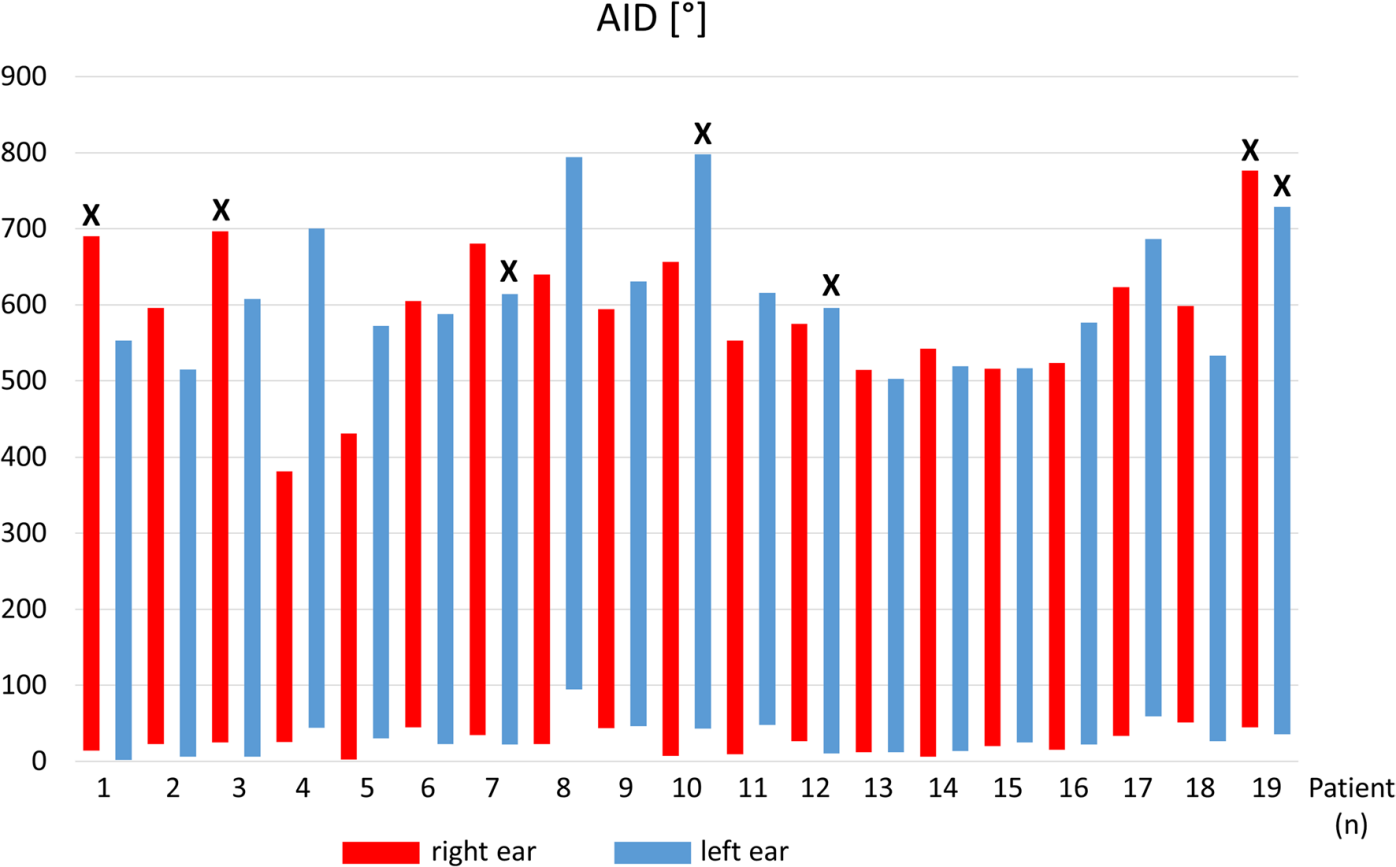
Distribution of insertion angles. Each bar represents the insertion angles for the most apical and most basal electrode of the individual patients for the right (red) and left (blue) ear. Crosses above the bars indicate Standard electrode arrays. Bars without crosses above them represent cases with flex28 electrode arrays

We further analysed the AID of all inserted electrodes comparing the two different electrode types. As the AID of the most apical electrode ranged from 381.6° to 798.0° (mean_Standard_ = 663.1°; SD_Standard_ = 125.7°) in the Standard electrode cohort and from 430.8° to 784.2° (mean_flex28_ = 581.3°; SD_flex28_ = 71.1°) in the flex28 electrode cohort, the Standard electrode was inserted significantly more deeply than the flex28 electrode (*p* = 0.018), corresponding to the greater length of the electrode array. Figure 2 shows the insertion range for each ear (defined as the range of the insertion angle from the most apical to the most basal electrode).

The comparison of the average AID of the most apical electrode between the two ears of the subjects did not reveal significant differences among the entire study collective. When analysing the AIDs of each patient individually, three cases showed significant differences. One of the three subjects showed electrode kinking on the side with less deep insertion (patient 4, right ear). The patient concerned showed better long-term hearing results on the side with electrode kinking compared to the other side (WRS_65_[CI, long-term]_right_ = 30%; WRS_65_[CI, long-term]_left_ = 5%; perilingually deafened patient). However, in the case of prelingual deafness and overall poor performance, no conclusions can be drawn here. The two other subjects showed similar results in the long-term speech perception.

**Fig. 3:**
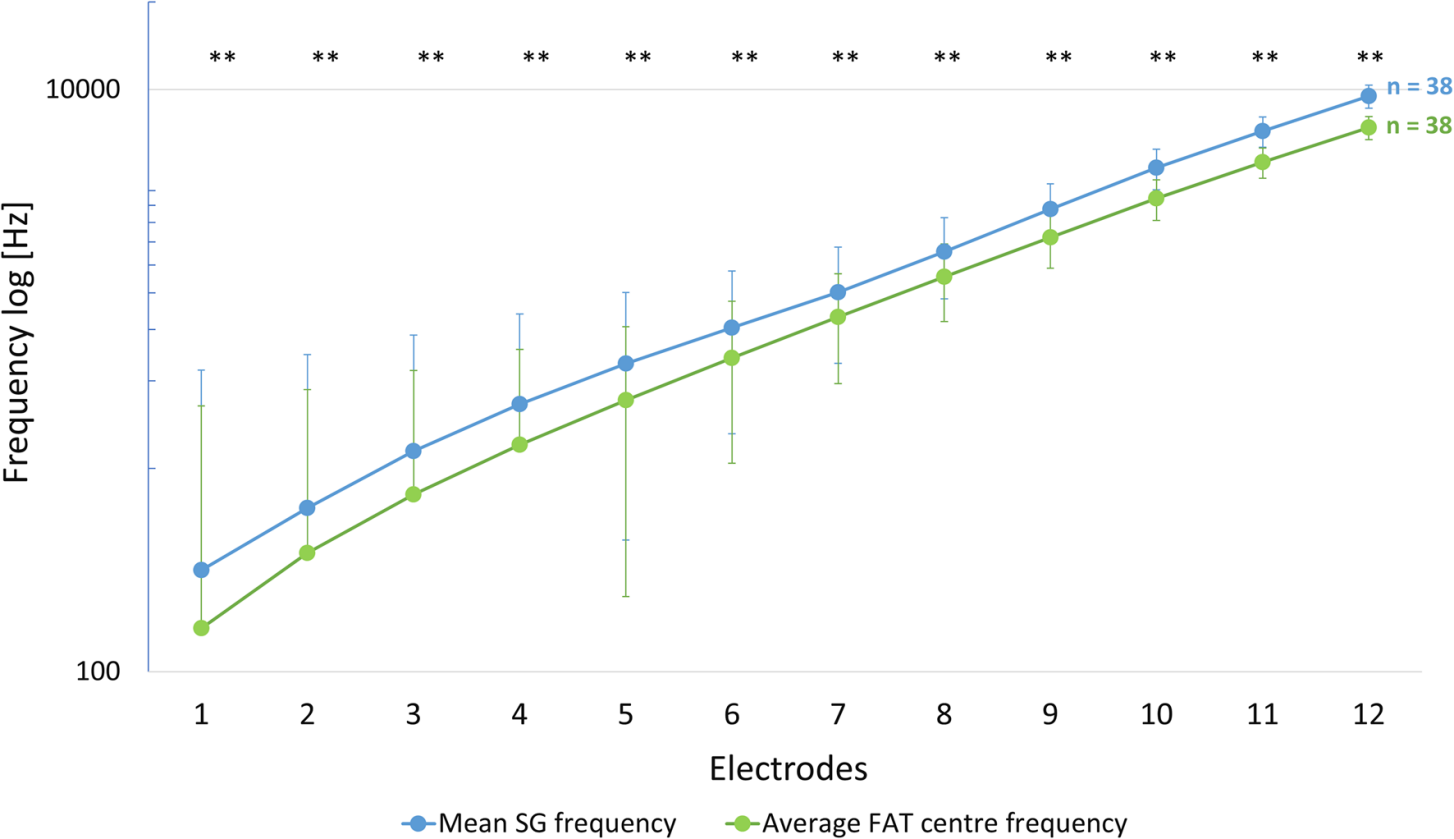
Comparison of SG map frequencies with fitted FAT frequencies. Comparison between distributions of frequencies at the SG map (spiral ganglion map) for the entire study cohort with the FAT (frequency allocation table) frequencies of the fitted map (logarithmic representation). There were significant differences (**) between mean SG frequency and average FAT centre frequency for every single electrode (*p* < 0.001)

Concerning FPM (Fig. 3), we found very significant differences between frequencies on the SG map and FAT frequencies, both on average and for each electrode individually (*p* < 0.001, Wilcoxon test for related samples). The frequencies on the SG map were on average 1.33 times as high as the FAT centre frequencies. The side comparison showed no significant differences for SG map frequencies (*p* = 0.746) and FAT frequencies (*p* = 0.290) between the right and left ear (Median test for independent samples).

To determine whether there was a connection between the average FPM for each ear individually and the corresponding speech perception/recognition among the entire collective, we calculated the Spearman’s correlation. One year after surgery there was neither a correlation for the electrodes 5–8 (*p* = 0.960) as the most relevant electrodes for the speech area (centre frequencies 836–2222 Hz), nor for all electrodes (1–12) (*p* = 0.710).

A significant effect of angular separation on speech perception was described before. It was suspected that larger angular separation allows a discrete stimulation of SG neuronal populations with consecutive improvement in the clarity of speech perception [11]. In our cohort, there was no significant correlation between angular separation in the 1–2 kHz region (about 224°–333°) as the most important region for speech recognition (*p* = 0.841, Spearman’s correlation). There was further no impact of gender on speech perception (*p* = 0.833, Mann–Whitney *U* test).

## Discussion

**Fig. 4:**
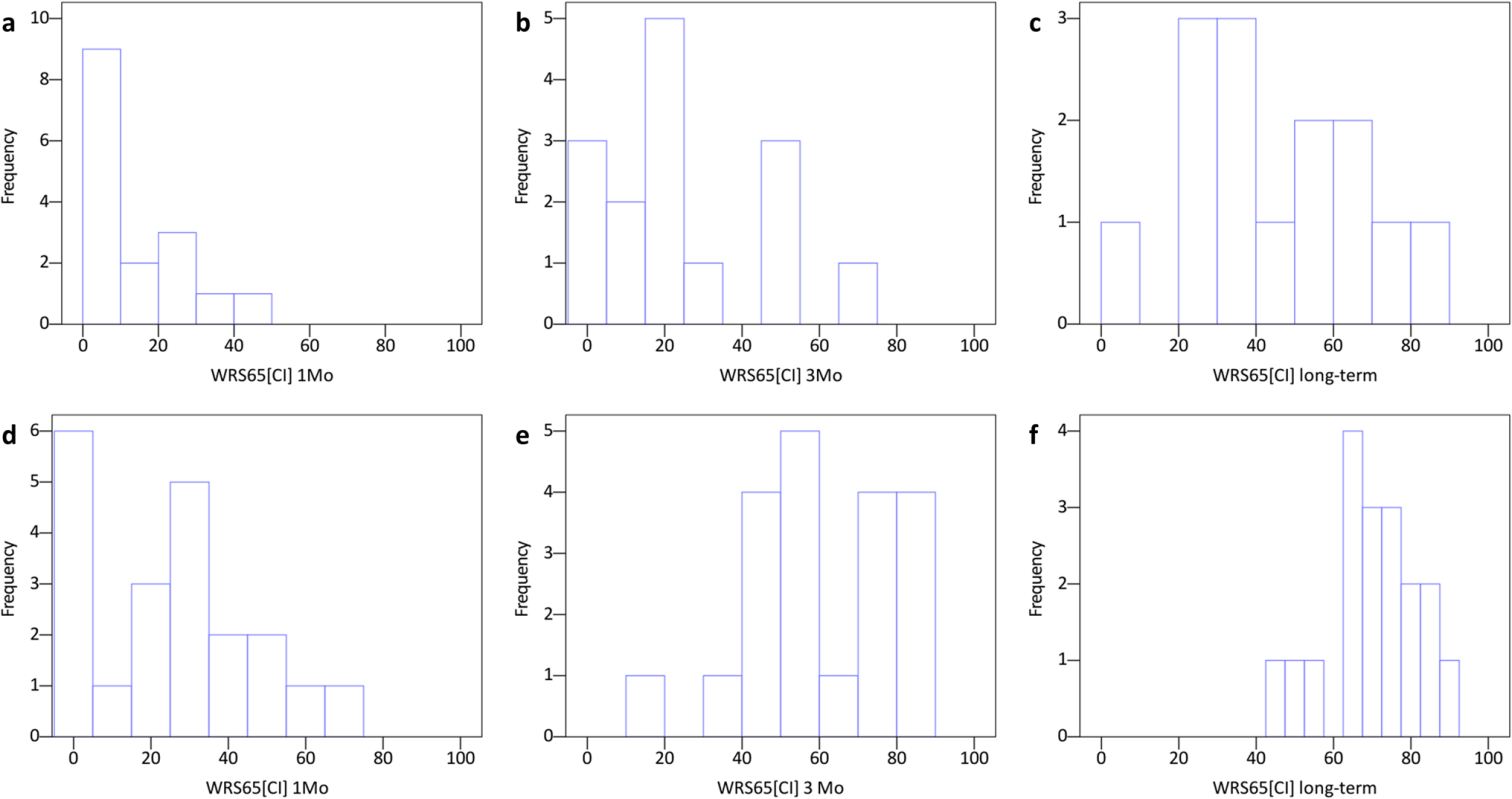
Speech perception in peri- and postlingually deafened subjects. Histograms in panels (**a**–**f**) display the non-normal distribution of WRS65[CI] (word recognition score at 65 dB with CI) Initial Score, WRS65[CI] after 3 months (**b**, **e**), and WRS65[CI] long-term Score (**c**, **f**) separately for peri- (**a**–**c**) and postlingually deafened patients (**d**–**f**)

The great variability in speech understanding among CI recipients is still a key issue today. Our study confirmed earlier investigations revealing varying results in speech perception at different postoperative points (Fig. 4a, b) [27]. The reasons for these highly different audiometric outcomes, despite apparently standardised conditions, have still not been clarified satisfactorily, although many studies are devoted to this topic. It has been proved that preoperative, residual speech comprehension is one essential component for the audiometric outcome in patients with postlingual profound hearing loss receiving a CI [28]. In addition, various other influencing factors, such as training effort, personal drive, neural survival or other patient-related factors, such as chronic diseases and intelligence, certainly play essential roles. As these factors can only be influenced to a limited extent, influenceable parameters affecting speech comprehension in CI recipient must be identified to optimise speech comprehension and to gain a better predictability of the individual outcome after cochlear implantation.

Based on improved, high-resolution imaging, more detailed examinations of anatomical and situational factors after CI are possible [24]. Therefore, using specific software programs, precise statements can be made on the exact frequency localisation within the cochlea of individual patients and thus also on the frequency-specific position of CI electrodes [6, 25]. The possible influence of location-related factors despite very standardised electrodes/surgical techniques was initially suspected because of the variable morphology of the cochlea with different CDLs and different numbers of turns [1–4]. We, were able to determine significant differences in the CDL between peri- and postlingually deaf patients with a morphologically normal cochlear anatomy (*p* = 0.045), with a CDL ranging from 32.79 to 44.51 mm.

In addition to individual anatomical differences, parameters that are influenced by the electrode position were also compared regarding their potential impact on the audiometric outcome of CI patients. One of these parameters, which can be easily examined by means of diagnostic imaging, is the insertion depth of the electrodes. Since there are various different electrode types with different lengths and designs, only lateral wall electrodes were considered in this study for better comparability. Deeper insertion depth of lateral wall electrodes has been described as a positive influencing factor on speech understanding [7, 8, 15, 16]. Yet, other studies negate this influence or even describe a negative influence on the audiometric outcome of the affected patients [9, 17–19]. Among our study cohort we found a significant difference in the AID between patients receiving the Standard electrode and the flex28 electrode corresponding to the greater length of the electrode (*p* = 0.018). However, we could not prove any significant influence of the AID on speech perception. Even intra-individual side-by-side comparison of the three patients with significantly different AIDs on both sides revealed no advantage in the deeper insertion depth. On the contrary, one of these patients heard significantly better in the ear with less insertion depth.

Another factor influencing patient outcome after CI implantation is the FPM. Due to the incomplete coverage of the entire cochlear length, the frequency-specific stimuli of the individual electrodes do not match the frequency location [10]. Therefore, the insertion depth of the electrode influences the FPM. Regarding FPM and speech perception there are likewise controversial results in the literature. Some relevant studies pointed out a significant relationship between speech recognition and FPM [10, 29], others, in turn, found no significant correlation [11]. In the course of our study we could not find any significant connection between FPM and speech comprehension, neither initially (*p* = 0.568) nor long-term (*p* = 0.960). Theories stating that FPM can be compensated by neural adaptation mechanisms in the long term [30]. Therefore, CI patients can generally tolerate a shift of ± 3 mm in the place of stimulation with only slight decrements in speech perception [31, 32]. Since we did not find any significant difference in the intra-individual side-by-side comparison, despite a very significant frequency to place mismatch (Fig. 3), the above-mentioned assumptions correspond with our results and suggest that proper place pitch matches might not be critical for basic speech recognition.

Another important factor is the so-called angular separation. It is assumed that, due to the different turns of the cochleae and the different individual insertion depths, the electrode contacts, which are attached to the array at fixed intervals, show varying degrees of angular separation of the neighbouring stimulated neuronal populations. A greater angular separation is thought to lead to a more specific stimulation of frequency-specific SG neuronal populations, which in turn can improve the clarity of speech recognition. A smaller angular separation, on the other hand, could lead to larger overlaps of the neural stimulation [33, 34]. Canfarotta et al. [11] showed that angular separation in the range of 1–2 kHz (about 224°–333°) has a significant influence on both the CNC words in quiet (*p* = 0.026) and the HINT sentences in noise (*p* = 0.018). In our patient cohort these results could not be reproduced for WRS_65_[CI] in the 1–2 kHz region (about 224°–333°) as the most important region for speech recognition (*p* = 0.841).

Many studies mentioned above share the same limitations: First, the inclusion of electrodes with different designs from different manufacturers were compared. Second, only different unilaterally implanted postlingually patients with profound hearing loss were compared with consecutive non-assesability of the effect of individual influences. In contrast, studies with bilaterally implanted patients are rare [14]. Thus, the strength of our study lies in examining patients with both peri- and postlingual profound hearing loss and bilateral cochlear implantation of lateral wall electrodes from only one manufacturer regarding the different anatomical and electrode-dependent parameters. Thanks to the bilateral implantation, an intra-individual comparison was possible within the scope of our study to attach less importance to patient-related or side-related ("ear effect") factors.

Although there are studies which simulate different insertion depths within an individual by inactivating electrodes [7, 35], the influence of the missing information from the inactivated electrodes cannot be assessed.

## Conclusion

There were no significant correlations between AID, FPM or angular separation regarding the audiometric outcome by means of an intra-individual and inter-individual comparison of bilaterally implanted patients with lateral wall CI electrodes—although the audiometric outcome varied significantly between individuals in our cohort. Therefore, the exact electrode position seems to have less influence on the speech recognition of CI patients than individual, already established parameters as preoperative speech recognition or duration of deafness.

## Supplementary Information

Below is the link to the electronic supplementary material.

Supplementary file1 (DOCX 14 KB)

## Data Availability

The authors confirm that the data supporting the findings of this study are available within the article and its supplementary materials.
