# Supplementary material for: Association between lateral wall electrode array insertion parameters and audiological outcomes in bilateral cochlear implantation
Source: Eur Arch Otorhinolaryngol. 2022 Nov 27;280(6):2707–14. doi: 10.1007/s00405-022-07756-2 (PMC10175364; doi:10.1007/s00405-022-07756-2)
Supplement: Supplementary file 1 — Supplementary file1 (DOCX 14 KB) [file 405_2022_7756_MOESM1_ESM.docx]

**Supplementary Material**

**Patient Data**

| **Patient** | **Gender** | **Side** | **Age at surgery** | **Onset of Deafness** | **Cause of Deafness** | **Electrode Type** |
| --- | --- | --- | --- | --- | --- | --- |
| **n1** | female | right  left | 71  75 | postlingual | unknown | Standard  Flex28 |
| **n2** | female | right  left | 75  74 | postlingual | unknown | Flex28  Flex28 |
| **n3** | female | right  left | 63  65 | postlingual | unknown | Standard  Flex28 |
| **n4** | male | right  left | 60  60 | perilingual | unknown | Standard  Standard |
| **n5** | male | right  left | 64  65 | postlingual | unknown | Flex28  Flex28 |
| **n6** | male | right  left | 64  63 | perilingual | Meningitis | Flex28  Flex28 |
| **n7** | female | right  left | 58  56 | perilingual | Meningitis | Flex28  Standard |
| **n8** | female | right  left | 60  56 | perilingual | family-related | Flex28  Flex28 |
| **n9** | female | right  left | 60  61 | perilingual | unknown | Flex28  Flex28 |
| **n10** | female | right  Left | 55  48 | postlingual | unknown | Flex28  Standard |
| **n11** | male | right  left | 52  56 | perilingual | family-related | Flex28  Flex28 |
| **n12** | male | right  left | 50  49 | perilingual | unknown | Flex28  Standard |
| **n13** | female | right  left | 51  50 | perilingual | unknown | Flex28  Flex28 |
| **n14** | female | right  left | 56  52 | perilingual | Menignitis | Flex28  Flex28 |
| **n15** | male | right  left | 52  51 | postlingual | unknown | Flex28  Flex28 |
| **n16** | female | right  left | 46  48 | postlingual | whole brain irradiation | Flex28  Flex28 |
| **n17** | female | right  left | 42  43 | perilingual | unknown | Flex28  Flex28 |
| **n18** | female | right  left | 34  34 | perilingual | unknown | Flex28  Flex28 |
| **n19** | female | right  left | 18  19 | perilingual | menignitis | Standard  Standard |
